# Association between the Presence of Autoantibodies against Adrenoreceptors and Severe Pre-Eclampsia: A Pilot Study

**DOI:** 10.1371/journal.pone.0057983

**Published:** 2013-03-04

**Authors:** Guiling Ma, Yanfang Li, Juan Zhang, Hao Liu, Dongyan Hou, Lei Zhu, Zhenyu Zhang, Lin Zhang

**Affiliations:** 1 Heart Centre, Capital Medical University, Chao-Yang Hospital, Beijing, China; 2 Gynaecology and Obstetrics Department, Capital Medical University, Chao-Yang Hospital, Beijing, China; Glaxo Smith Kline, Denmark

## Abstract

**Background:**

Pre-eclampsia is the leading cause of maternal and neonatal morbidity and mortality with incompletely understood etiopathogenesis. The purpose of the current study is to determine whether there is a relationship between the presence of autoantibodies against β_1_, β_2_ and α_1_ adrenoreceptors and severe pre-eclampsia.

**Methodology/Principal Findings:**

Synthetic peptides corresponding to amino acid sequences of the second extracellular loops of β_1_, β_2_ and α_1_ adrenoreceptors were synthesized as antigens to test 34 patients with severe pre-eclampsia, 36 normal pregnancy women and 40 non-pregnant controls for the presence of autoantibodies using enzyme-linked immunosorbent assay. The respective frequencies of autoantibodies against β_1_, β_2_ and α_1_ adrenoreceptors were 50.0% (17/34), 52.9% (18/34) and 55.9% (19/34) in patients with severe pre-eclampsia, 19.4% (7/36) (p = 0.011), 19.4% (7/36) (p = 0.006) and 17.6% (6/36) (p = 0.001) in normal pregnancy women and 10% (4/40), 7.5% (3/40) and 10% (4/40) (p<0.001) in non-pregnant controls. Titers of these autoantibodies were also significantly increased in patients with severe pre-eclampsia. By logistic regression analysis, the presence of these three autoantibodies significantly increased the risk of neonatal death (odds ratio, 13.5; 95% confidence interval, 1.3–141.3; p = 0.030) and long-term neonatal hospitalization (odds ratio, 5.0; 95% confidence interval, 1.3–19.1; p = 0.018). The risk of hypertension and fetal distress were also associated with the presence of these three autoantibodies.

**Conclusions/Significance:**

This novel pilot study demonstrated for the first time that the presence of autoantibodies against β_1_, β_2_ and α_1_ adrenoreceptors are increased in patients with severe pre-eclampsia. Pregnant women who are positive for the three autoantibodies are at increased risks of neonatal mortality and morbidity. We posit that these autoantibodies may be involved in the pathogenesis of severe pre-eclampsia.

## Introduction

Pre-eclampsia is a serious hypertensive disorder during pregnancy that affects 3%-5% of pregnancies, and remains the leading cause of maternal and neonatal mortalities and morbidities in the world [1], [2]. It is a multi-systemic disease with features such as hypertension and proteinuria [3]. In serious cases, termination of pregnancy is the only available option to prevent further health deterioration of the fetus and mother [4]. To date, the factors and mechanisms involved in the pathogenesis of pre-eclampsia remain poorly understood.

Studies have described the role of autoantibodies against α_1_ adrenoreceptor (anti-α_1_-AR) in primary and malignant hypertension [5], [6]. Previously, we demonstrated that the presence of autoantibodies against β_1_, β_2_, and α_1_ adrenoreceptors (anti-β_1_, β_2_, and α_1_-ARs), which bind to the second extracellular loop of the receptors, are highly prevalent in hypertensive heart disease and may participate in its pathogenesis [7], [8]. In recent years, evidence has accumulated that suggests that autoimmunity participates in the pathogenesis of pre-eclampsia. Recently, numerous studies have shown that pre-eclamptic women possess autoantibodies against angiotension II type 1 receptor, which bind to and activate the receptor, consequently provoking biological responses relevant to the pathogenesis of pre-eclampsia [9]–[13]. The aim of this study was to investigate differences in the frequencies of anti-β_1_, β_2_, and α_1_-ARs among patients with severe pre-eclampsia, compared to normal pregnancy women and non-pregnant controls. The second aim was to investigate the relationship between the presence of anti-β_1_, β_2_, and α_1_-ARs and perinatal mortality and morbidity. We used synthetic peptides corresponding to amino acid sequences of the second extracellular loop of the human β_1_, β_2_, and α_1_ ARs, to test sera from patients with severe pre-eclampsia, normal pregnancy women, and non-pregnant controls.

## Results

Study subjects were enrolled from May 2011 to November 2011. Clinical characteristic of women from three study groups are shown in Table 1.

**Table 1:** Clinical characteristic of women from three groups in the present study.

	Non-pregnant	Normal pregnancy	Severe pre-eclampsia
	(n = 40)	(n = 36)	(n = 34)
Age (years)	30.4±3.9	29.6±3.3	31.3±4.7
Gestational age	NA	38.4±1.7	33.1±3.4*
Systolic blood pressure (mmHg)	118.7±6.8	115.2±6.9	166.5±17.2*
Diastolic blood pressure (mmHg)	74.7±6.3	73.0±5.7	105.0±11.4*
Urinary protein (mg/24 h)	Nd#	Nd#	3062.7±2538.5

Mean ± SD are shown. Student’s unpaired two-tailed t-test was made between non-pregnant group versus normal pregnancy group and normal pregnancy group versus severe pre-eclamptic group, significant differences (p<0.001) are indicated by*.

Nd: not determined; NA: not applicable.

# Urine protein of normal pregnancy and non-pregnant women was within normal ranges and not routinely recorded.

### Maternal Clinical Characteristics

Maternal hospital stay was significantly longer in the severe pre-eclampsia group than in the normal pregnancy group (8.9±1.1 days versus 4.5±0.5 days, p<0.001). Complications of pregnancy, such as placental abruption, placenta remnants and postpartum hemorrhage, were significantly higher in the severe pre-eclampsia group than in the normal pregnancy group (7/34 versus 0/36, p = 0.004).

### Perinatal Clinical Characteristics

The proportion of fetuses in the severe pre-eclampsia group that suffered from fetal growth restriction (58.8% (20/34)) and fetal distress (29.4% (10/34)), respectively, was significantly higher than 2.8% (1/36) and 8.33% (3/36) in the normal pregnancy group (p both <0.05). Preterm birth and low birth weight were significantly increased in the severe pre-eclampsia group compared with the normal pregnancy group (64.7% versus 11.1%, 76.5% versus 5.6%, p<0.001, respectively). Perinatal death was also increased in the severe pre-eclampsia group (11.8% versus 0%, p = 0.021) (Table 2).

**Table 2:** Perinatal complications.

Complications	Severe pre-eclampsia (%)	Normal pregnancy (%)	p value
Fetal growth restriction	20(58.8)	1(2.8)	<0.001**
Fetal distress	10(29.4)	3(8.3)	0.032*
Low birth weight	26(76.5)	2(5.6)	<0.001**
<1500 g	15(44.1)	0	<0.001**
<1000 g	4(11.8)	0	0.021*
Preterm	22(64.7)	4 (11.1)	<0.001**
<34 weeks	19(55.9)	0	<0.001**
Neonatal asphyxia	7(17.7)	0	0.001*
Mild	5(14.7)	0	0.008*
Severe	2(5.9)	0	0.150
Perinatal death	4(11.8)	0	0.021*
Intrauterine death	3(8.8)	0	0.056
Neonatal death	1(2.9)	0	0.391

* p<0.05;

** p<0.001.

**Figure 1:**
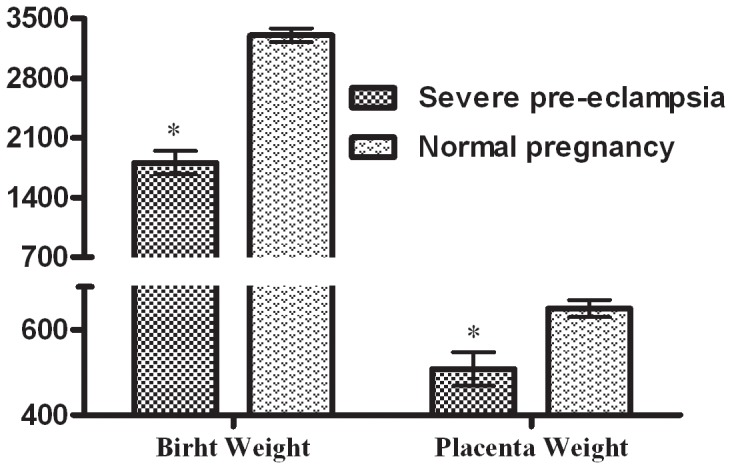
Birth weight and placenta weight. The birth weight of neonates in severe pre-eclamptic women was significantly lower than that in normal pregnancy women (1807.3±137.7 g versus 3279.2±65.9 g, p<0.001). Placenta weight was significantly reduced in severe pre-eclamptic women (507.9±39.6 g versus 649.6±20.1 g, p = 0.002).

Birth weight in the severe pre-eclampsia group was significantly lower than in the normal pregnancy group (1807.3±137.7 g versus 3279.2±65.9 g, p<0.001). Similarly, placental weight was also reduced in the severe pre-eclampsia group (507.9±39.6 g versus 649.6±20.1 g, p = 0.002, Figure 1).

**Figure 2:**
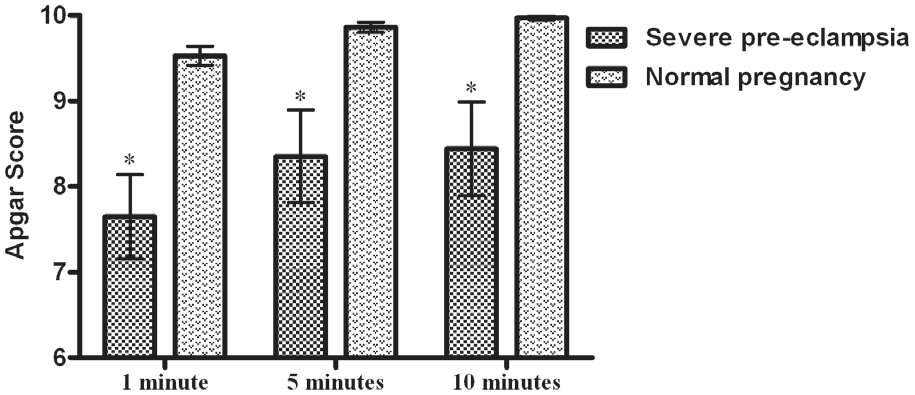
Apgar scores of neonates. The Apgar scores were significantly lower in severe pre-eclamptic women than in normal pregnancy women. The scores were: at one minute (7.7±0.5 versus 9.5±0.1, p<0.0001), five minutes (8.4±0.5 versus 9.9±0.06, p<0.0001) and ten minutes (8.4±0.6 versus 10.0±0.03, p<0.0001).

Neonatal Apgar score (includes assessment of the appearance of skin color, pulse, grimace, activity, and respiration) was used to classify or grade newborn infants [14]. The Apgar scores were significantly lower in the severe pre-eclampsia group than in the normal pregnancy group at one minute (7.7±0.5 versus 9.5±0.1, p<0.001), five minutes (8.4±0.5 versus 9.9±0.06, p<0.001), and ten minutes (8.4±0.6 versus 10.0±0.03, p<0.001) (Figure 2). Neonatal asphyxia was defined according to the Apgar score at one minute: mild was between 4 and 7 and severe was between 0 and 3. The incidence of neonatal asphyxia was significantly higher in the severe pre-eclampsia group than in the normal pregnancy group (17.7% versus 0%, p = 0.001).

There were different outcomes for the 34 neonates in the severe pre-eclampsia group: 11.8% (4/34) of infants died; 23.5% (8/34) of infants were transferred to the BaYi Children’s Hospital, affiliated to the military general hospital of Beijing PLA; 41.2% (14/34) of infants were transferred to the pediatric department of our hospital; 23.5% (8/34) did not require further treatment. The mean hospital stay for the neonates was 28.4±4.6 days.

### ELISA Results

**Figure 3:**
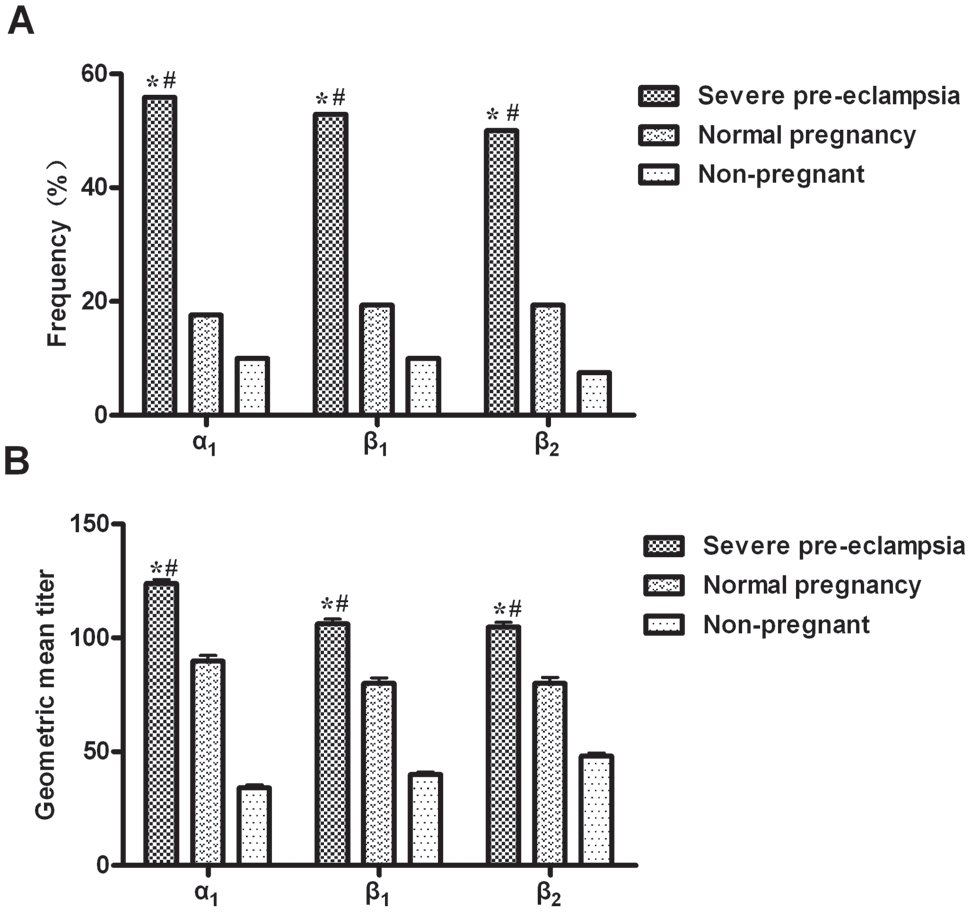
Frequencies and titers of autoantibodies among the three groups. Frequencies and geometric mean titers of anti-β_1_, β_2_, and α_1_-ARs were significantly higher in the severe pre-eclamptic women than in the normal pregnancy women and non-pregnant controls. ^#^: p<0.05 severe pre-eclamptic women compared to normal pregnancy women; *: p<0.001 severe pre-eclamptic women compared to non-pregnant controls.

Sera positive for anti-β_1_-AR was found in 50.0% (17/34) of the severe pre-eclampsia group, 19.4% (7/36) (p = 0.011) of the normal pregnancy group, and 10.0% (4/40) (p<0.001) of non-pregnant controls. Sera positive for anti-β_2_-AR was found in 52.9% (18/34) of the severe pre-eclampsia group, 19.4% (7/36) (p = 0.006) of the normal pregnancy group, and 7.5% (3/40) (p<0.001) of non-pregnant controls. Positive sera for anti-α_1_-AR was found in 55.9% (19/34) of the severe pre-eclampsia group, 16.7% (6/36) (p = 0.001) in the normal pregnancy group, and 10.0% (4/40) (p<0.001) of non-pregnant controls. Titers of the anti-β_1,_ β_2_ and α_1_-ARs were significantly higher in the severe pre-eclampsia group than in the other two groups (p<0.001), Figure 3.

Positive sera from the severe pre-eclampsia group contained different kinds of autoantibodies. Twelve patients were positive for a single autoantibody, four patients were positive for two kinds of autoantibody, and 11 patients were positive for all the three kinds of autoantibody. A significant correlation was found between anti-β_1_-AR and anti-α_1_-AR (*r_s_* = 0.4, p = 0.02), anti-β_1_-AR and anti-β_2_-AR (*r_s_* = 0.6, p<0.001). In the severe pre-eclampsia group, 76.5% (13/17) of the 17 women with anti-β_1_-AR, were also positive for anti-α_1_-AR, and 82.4% (14/17) had anti-β_2_-AR.

### The Association of Autoantibody and Clinical Outcomes

Univariate logistic regression assessed the association of autoantibody with hypertension (≥160 mmHg systolic or ≥110 mmHg diastolic), pregnancy complications, fetal growth restriction, fetal distress, preterm birth, low birth weight, neonatal long-term hospitalization (more than two weeks), and perinatal death. The presence of one or two kinds of autoantibody against β_1_, β_2_, and α_1_ ARs had no statistical relationship with any complication. In contrast, the risks for both maternal and perinatal complications were closely associated with the presence of the three autoantibodies. Positivity for anti-β_1_, β_2_, and α_1_-ARs was associated with hypertension (OR, 6.5; 95% CI, 1.6–25.7; p = 0.008), pregnancy complication (OR, 4.1; 95%CI, 1.0–16.0; p = 0.044), fetal growth restriction (OR, 3.7; 95%CI, 1.1–12.0; p = 0.032), fetal distress (OR, 6.0; 95% CI, 1.7–21.7; p = 0.006), preterm birth (OR, 4.9, 95%CI, 1.4–16.5; p = 0.011), low birth weight (OR, 4.5; 95%CI, 1.3–15.1; p = 0.016), perinatal death (OR, 13.5; 95% CI, 1.3–141.3; p = 0.030), and long-term neonatal hospitalization (OR, 5.0; 95% CI, 1.3–19.1; p = 0.018).

## Discussion

### Main Findings

In this study, we demonstrated for the first time that positivity for anti-β_1_, β_2_, and α_1_-ARs was associated with severe pre-eclampsia. The frequencies and titers of anti-β_1_, β_2_, and α_1_-ARs were significantly higher in women with severe pre-eclamptic, when compared to normal pregnancy women and non-pregnant healthy controls. The presence of the three autoantibodies was associated with both adverse maternal and perinatal clinical outcomes including hypertension, pregnancy complications, fetal growth restriction, fetal distress, preterm birth, low birth weight, perinatal death, and long-term hospitalization of neonates.

### Immune Mechanisms in Pre-eclampsia

The pathogenesis of pre-eclampsia remains obscure, but is likely multifactorial involving abnormal placentation, reduced placental perfusion, endothelial cell dysfunction, and systemic vasospasm [15]. An immune mechanism has long been postulated in the pathogenesis of pre-eclampsia. Immune maladaptation may impair invasion of spiral arteries by endovascular cytotrophoblast cells [16]. Studies have suggested that repeated exposure to sperm from a particular male partner prior to pregnancy promotes immune tolerance and reduces the risk of defective trophoblast invasion [17]. Autoantibodies, such as anticardiolipin and anti-β_2_-glycoprotein-1 antibody, have been detected in pre-eclampsia patients [18]. From the first report that described the presence of autoantibody against angiotensin II type 1 receptor in pre-eclampsia patients [9], researchers have gained a greater understanding on the pathogenic mechanisms underlying pre-eclampsia which implicate the immune system.

### Autoantibodies and Pre-eclampsia

While our results need to be confirmed by larger studies, there are biologically plausible mechanisms by which anti-β_1_, β_2_, and α_1_-ARs may lead to severe pre-eclampsia. The β_1_ and β_2_ adrenoreceptors in the human heart couple to the G protein Gs, which activates adenylyl cyclase. Stimulation of both receptor subtypes increases the intracellular level of cAMP, which leads to phosphorylation of target proteins [19]. α_1_ adrenoreceptor couple predominantly via Gq, resulting in hydrolysis of membrane phospholipids via phospholipase C β, to yield the second messengers inositol triphosphate and diacylglycerol. This leads to muscle contraction through mobilization of intracellular Ca^2+^ [20] and activation of protein kinase C. Frequencies and titers of anti-β_1_, β_2_, and α_1_-ARs were significantly increased in the severe pre-eclampsia group than in the normal pregnancy women and non-pregnant controls. Therefore we posit that there may be a relationship between the presence of anti-β_1_, β_2_, and α_1_-ARs and the development of severe pre-eclampsia. Alternatively, it is plausible that severe pre-eclampsia triggers the production of anti-β_1_, β_2_, and α_1_-ARs. Further studies are needed to delineate these two possible pathways.

Studies have shown that the risk of long-term sequelae such as chronic hypertension, ischemic heart disease, stroke, and venous thromboembolism are significantly increased in pre-eclamptic women [21], [22]. In this study, we were able to collect blood samples from six of the 34 patients with severe pre-eclampsia at the end of puerperium without scheduled follow-up. Three of the six samples were positive for anti-β_1_, β_2_, and α_1_-ARs at similar titers to levels at recruitment. This is but distinct from what has been observed for autoantibodies against angiotension II type 1 receptor which have been shown to subside after pregnancy termination [9]. We infer that the presence of autoantibody might be related to high risk for cardiovascular sequelae, but this hypothesis remains to be further explored.

Three kinds of autoantibodies closely related to each other were detected in the severe pre-eclampsia group. Approximately 32.4% (11/34) of severe pre-eclamptic women had all three kinds of autoantibodies. This indicates that the autoimmune response in severe pre-eclamptic patients is multi-faceted.

### Conclusion

This pilot study has demonstrated for the first time that the presence of anti-β_1_, β_2_, and α_1_-ARs were prevalent in a cohort of severe pre-eclamptic women. Risks of both maternal and perinatal complications were significantly increased when all the three kinds of autoantibody studied were present. We posit that autoantibodies against adrenoreceptors may be involved in the pathogenesis of severe pre-eclampsia. Further studies are needed to confirm these findings and dissect the underlying mechanisms for this novel observation.

## Materials and Methods

### Ethics Statement

The research protocol was conducted in accordance with the guidelines of the World Medical Association’s Declaration of Helsinki and was performed following approval from the Medical Ethics Committee (12–S-63) of Capital Medical University Beijing Chao-Yang Hospital. All pregnant women were included in the study during the prepartum or early intrapartum period and provided written informed consent before inclusion in the study.

### Subjects

Patients who were admitted to Beijing Chao-Yang Hospital were managed by the obstetrics faculty of the Capital Medical University. Thirty-four patients were diagnosed with severe pre-eclampsia based on the criteria set by the National High Blood Pressure Education Program Working Group report [23]. The criteria include increased blood pressure (≥160 mmHg systolic or ≥110 mmHg diastolic on two occasions at least 6 hours apart) after 20 weeks of gestation in women with previously normal blood pressure or proteinuria of ≥2 g/24 h. Patients with severe pre-eclampsia and normal pregnancy women were approached for the study during the antepartum or early intrapartum period. We selected two comparison groups: 36 apparently healthy pregnant women and without hypertension or proteinuria (normal pregnancy group) and 40 healthy non-pregnant women (non-pregnant group). Exclusion criteria for all groups were diabetes mellitus, vasculitis, renal disease and autoimmune disease. Blood samples were collected from antecubital vein at recruitment using tubes containing EDTA, and centrifuged at 2000×g for 10 minutes at 4°C within 2 h of the collection. Serum samples were stored at −70°C. We were able to collect blood samples from six patients with severe pre-eclampsia at the end of puerperium. Placentas were collected from study subjects and weighed.

We collected clinical data from mother and infants/neonates. In this study, low birth weight was defined as body weight less than 2500 g at birth. Gestational age less than 37 weeks was considered as preterm birth. Fetal growth restriction was defined as the failure to reach the predetermined growth potential. This was operationally defined as sonographic estimated fetal weight below the 10th percentile for their gestational age. A fetal heart rate ≥160 bpm or ≤110 bpm, evaluated by electronic fetal monitoring, or the third degree of meconium-stained amniotic fluid was considered evidence of fetal distress.

### Materials

Three peptides corresponding to the amino acid sequence of the second extracellular loop of human β_1_, β_2_, and α_1_ ARs were synthesized by Genomed (Genomed Synthesis, Inc., CA, USA) and the sequences are shown in Table 3 [24]–[26]. The purity of the peptides, determined by high performance liquid chromatography (HPLC) using a Vydac C-18 column, was above 95% as shown in Figure S1. The molecular weight of peptides was analyzed by mass spectrometry as shown in Figure S2. Nunc microtiter plates were purchased from Kastrup, Denmark. Tween-20, thimerosal, and ABTS were obtained from Sigma, St. Louis, MO, USA. Fetal bovine serum, biotinylated goat anti-human IgG (H+L), and horseradish peroxidase-streptavidin were bought from Zhongshan Golden Bridge Biotech, Beijing, China. The microplate reader was purchased from Molecular Devices Corp, Menlo Park, CA.

**Table 3:** Amino acid sequences of human β_1_, β_2_, and α_1_-adrenoreceptors.

Adrenoreceptor	Position	Sequence
β_1_	197-222	H-W-W-R-A-E-S-D-E-A-R-R-C-Y-N-D-P-K-C-C-D-F-V-T-N-R
β_2_	172-197	H-W-Y-R-A-T-H-Q-E-A-I-N-C-Y-A-N-E-T-C-C-D-F-F-T-N-Q
α_1_	192-218	G-W-K-E-P-V-P-P-D-E-R-F-C-G-I-T-E-E-A-G-Y-A-V-F-S-S-V

### ELISA Protocol

Samples were classified as positive or negative based upon the presence or absence of anti-β_1_-AR, anti-β_2_-AR, and anti-α_1_-AR. An ELISA protocol, previously described by Fu et al [27], was used to screen for the presence of the autoantibodies. Briefly, 50 µL of peptide (5 mg/L) in 100 mmol Na_2_CO_3_ solution (pH = 11) was coated on microtiter plates overnight at 4°C. The wells were saturated with PMT (1× PBS, 1 mL/L Tween-20, and 0.1 g/L thimerosal (PBS-T) supplemented with 100 mL/L fetal bovine serum) for 1 hour at 37°C. Then 50 µL of serum diluted from 1∶20 to 1∶160, positive control and negative control were added to the wells for 1 hour at 37°C. After washing the wells with PBS-T for three times, affinity-purified biotinylated goat anti-human IgG (H+L) (1∶500 dilution in PMT) was added for 1 hour at 37°C. Following another round of washing three times, the bound biotinylated antibody was detected by incubating the microtiter plate for 1 hour at 37°C with horseradish peroxidase-streptavidin (1∶500 dilution in PMT). This was followed by three times washing in PBS, the addition of 2.5 mmol/L H_2_O_2,_ and then of 2 mmol/L ABTS in citrate buffer solution. Absorbance (*A*) was measured after 30 minutes at 405 nm in a microplate reader.

### Data Analysis

Quantitative data were expressed as the mean ± SD. Positivity was defined as the ratio of (sample *A -* blank *A*)/(negative control *A* - blank *A*) ≥2.1. Antibody titer was reported as geometric mean. Data were analyzed using SPSS 16.0. (SPSS, Chicago, Illinois, USA) Fisher’s exact test and unpaired *t* tests were used to determine the significance in differences between groups. The correlation with autoantibodies was tested using the Spearman correlation coefficient. Association between the presence of autoantibodies and categorical outcomes was assessed using univariable logistic regression analysis. Tests with p<0.05 were considered statistical significant.

## Supporting Information

Figure S1 **Purity of the peptides.** The purity of the synthesized peptides corresponding to the amino acid sequence of the second extracellular loop of human β_1_, β_2_, and α_1_ adrenoreceptors, determined by HPLC, was 96.66%, 96.21% and 95.34%.
(TIF)

Figure S2 **Molecular weight of peptides.** Molecular weight of peptides corresponding to the amino acid sequence of the second extracellular loop of human β_1_, β_2_, and α_1_ adrenoreceptors was analyzed by mass spectrometry and the molecular weight was 3484.9, 3237.5 and 2872.2.
(TIF)
