# Supplementary figures and images for: Association between the Presence of Autoantibodies against Adrenoreceptors and Severe Pre-Eclampsia: A Pilot Study
Source: PLoS One. 2013 Mar 4;8(3):e57983. doi: 10.1371/journal.pone.0057983 (PMC3587423; doi:10.1371/journal.pone.0057983)

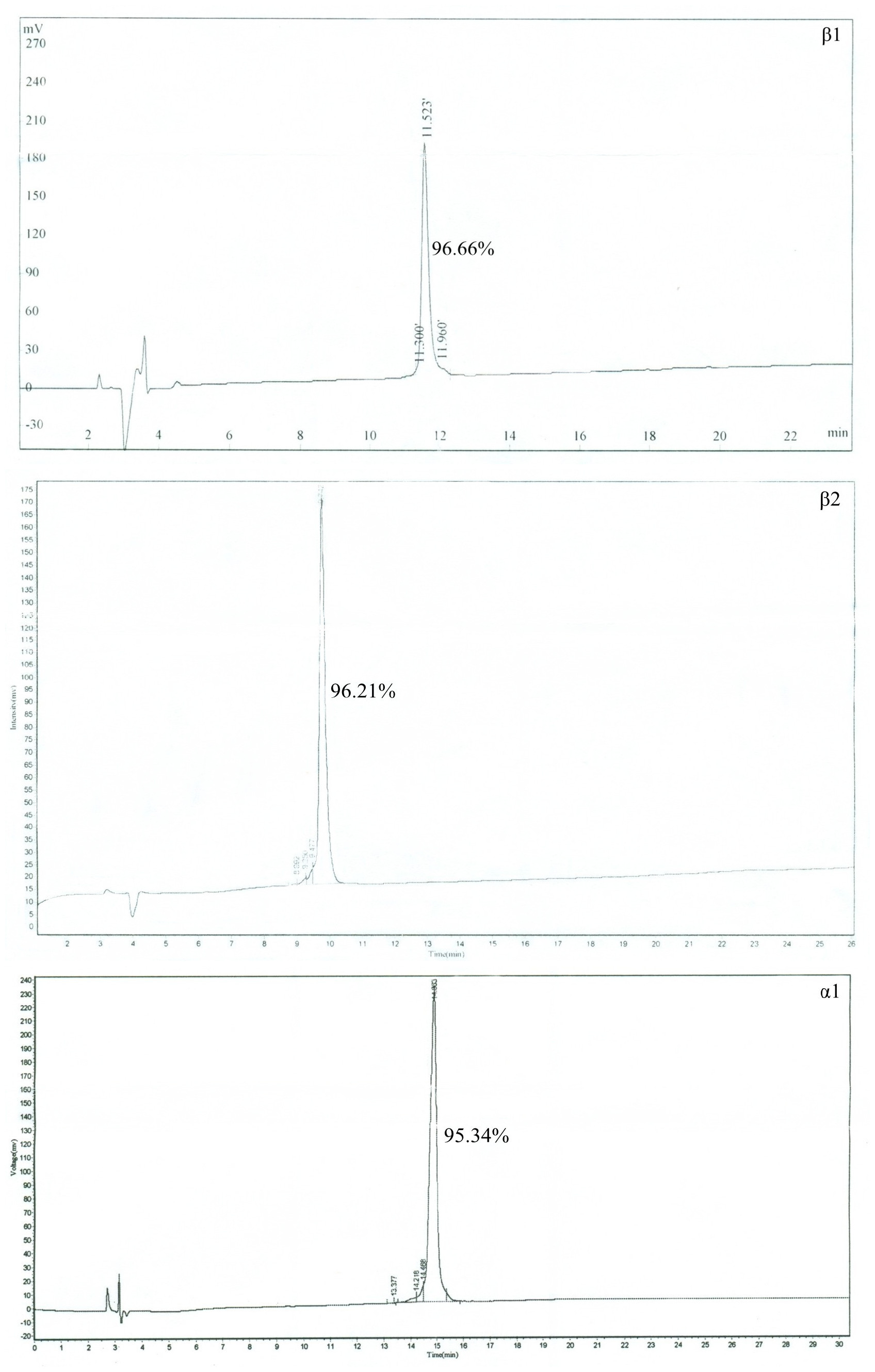

Supplement: Figure S1 — Purity of the peptides. The purity of the synthesized peptides corresponding to the amino acid sequence of the second extracellular loop of human β1, β2, and α1 adrenoreceptors, determined by HPLC, was 96.66%, 96.21% and 95.34%. (TIF) [file pone.0057983.s001.TIF]

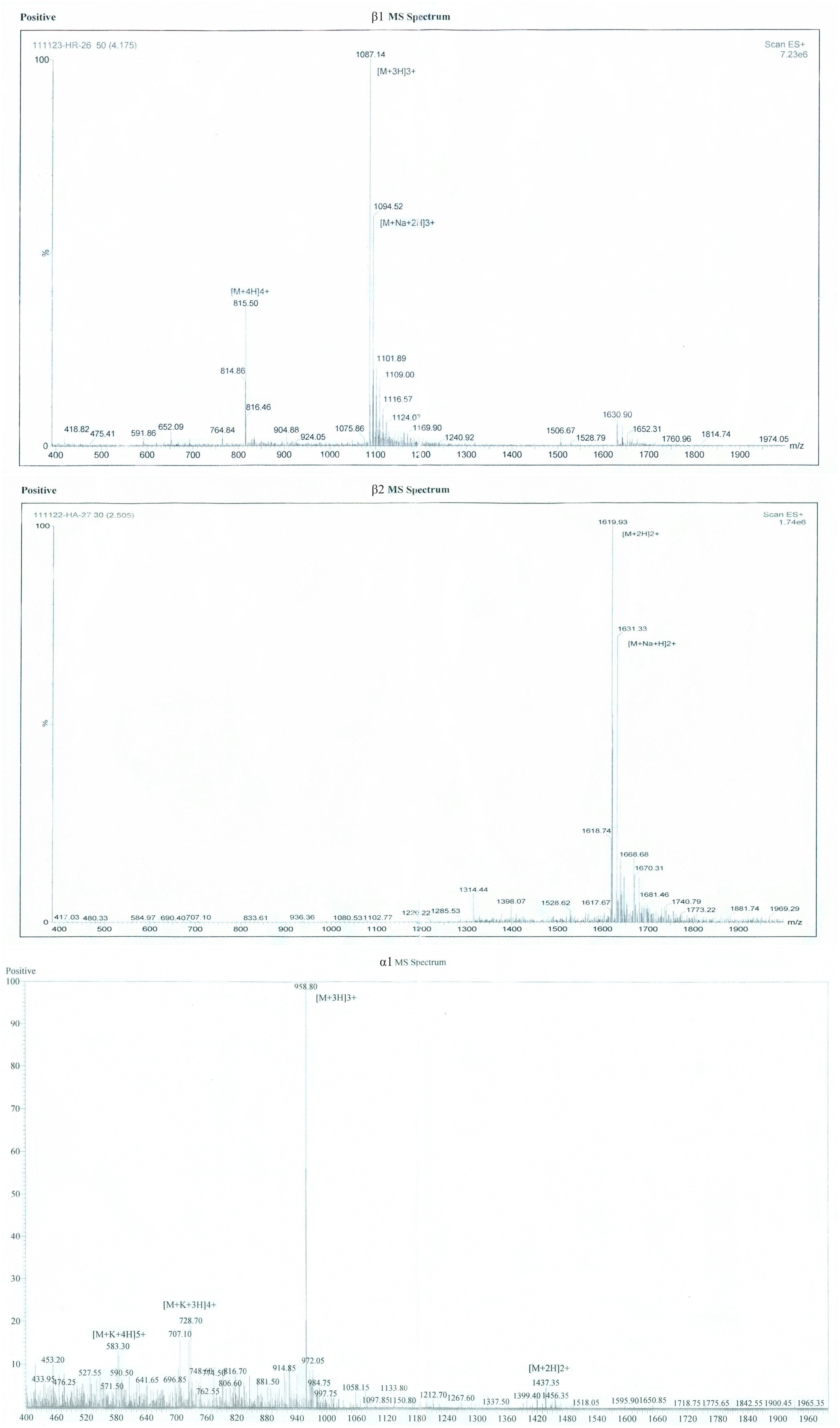

Supplement: Figure S2 — Molecular weight of peptides. Molecular weight of peptides corresponding to the amino acid sequence of the second extracellular loop of human β1, β2, and α1 adrenoreceptors was analyzed by mass spectrometry and the molecular weight was 3484.9, 3237.5 and 2872.2. (TIF) [file pone.0057983.s002.TIF]
